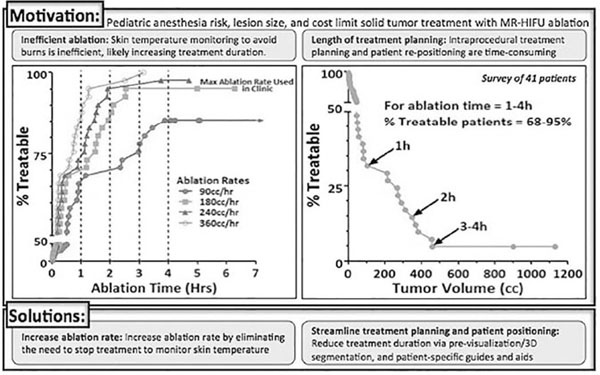# The optimization of treatment planning and ablation rate improvements on feasibility of pediatric MR-HIFU applications

**DOI:** 10.1186/2050-5736-3-S1-P77

**Published:** 2015-06-30

**Authors:** Doug Wackerle, Haydar Celik, David Kinnaird, Daniel Yang, Avinash Eranki, Matthew Oetgen, AeRang Kim, Karun Sharma, Harry Kim, Peter Kim, Pavel Yarmolenko

**Affiliations:** 1 The George Washington University School of Medicine, Washington, D.C., United States; 2 Children’s National Health System, Washington, D.C., United States; 3 Princeton University, Princeton, New Jersey, United States; 4 Texas Scottish Rite Hospital for Children, Dallas, Texas, United States

## Background/introduction

Magnetic resonance-guided high intensity focused ultrasound (MR-HIFU) ablation provides a precise, non-invasive treatment for lesions in adults. In children, MR-HIFU’s potential remains largely unexplored, though its non-invasive and non-ionizing nature holds promise. Yet, pediatric patients pose challenges affecting treatment: young children require general anesthesia, exhibit wide ranges of anatomy, and have varying lesion sizes and locations. These demonstrate a need for standardized treatment approaches and physical aids to optimize patient position, reduce time-intensive repositioning, and thus reduce overall treatment time. Further improvement of ablation rate and reduction of risk are also possible via improved monitoring of skin temperature during ablation and mild hyperthermia. Improvements in treatment planning and volumetric rate may save time and allow for treatment of larger lesions, increase patient throughput, and possibly increase efficacy and lower cost. This study aims to quantify and examine how such improvements could increase the time allocated for direct ablation and produce better outcomes.

## Methods

Forty-one pediatric patients with various limb tumors at Children’s National Medical Center from November 2005 to October 2013 were examined retrospectively as potential candidates for MR-HIFU ablation therapy. After identifying the tumor location, software (Avizo Standard Edition 8.0.0, Visualization Sciences Group, SAS, Berlin, Germany) was used to define its area through axial slices and create a 3D segmented model to measure its volume. As a reference, treatment time was estimated at a maximum (180 cc/hour) rate used in ablation of uterine fibroids (obtained from Phillips Healthcare, Vantaa, Finland).

Four hours maximum anesthesia time was selected due to risks to children and restraints on surgeon time and focus, room and machine time, and cost. Tumor volume and ablation rate data was graphically combined to show effects of theoretical improvements.

## Results and conclusions

Increasing the time available for ablation can substantially increase treatable tumor volume. In the examined 41 patients, utilizing only 1 hour for ablation (at 180 cc/hour) leaves 13 patients (32%) untreated. With more time, all but 2 patients (5%) are treatable with 3 or 4 hours of ablation. Conversely, complete treatment of a lesion is directly related to ablation rate. At the current rate of (180 cc/hour), 2 (5%) are untreatable, yet with double the current rate (360 cc/hour), all 41 lesions can be treated. Improvements in planning guidelines and treatment rates could have substantial impacts on the effectiveness of MR-HIFU ablation and the size of treatable tumors and number of patients treated with this technique.

**Figure 1:**